# An interprofessional model to improve LGBTQ+ specific cultural competence in dental and pharmacy students

**DOI:** 10.1371/journal.pone.0313492

**Published:** 2025-01-09

**Authors:** Anubhuti Shukla, Sriha Yalamanchi, Guillermo Tamayo-Cabeza, Amanda Albright, Zachary A. Weber, April D. Newton

**Affiliations:** 1 Department of Dental Public Health and Dental Informatics, Indiana University School of Dentistry, Indianapolis, IN, United States of America; 2 Indiana University Interprofessional Practice and Education Center, Indianapolis, IN, United States of America; 3 Purdue College of Pharmacy, Indianapolis, IN, United States of America; Far Eastern University - Manila, PHILIPPINES

## Abstract

**Background:**

Disproportionate access to healthcare services among the Lesbian, Gay, Bisexual, Transgender, Queer or Questioning and others (LGBTQ+) population can be partially attributed to the lack of cultural competence among healthcare providers. The aim of this study was to evaluate the impact of an interprofessional model in improving cultural competence and clinical preparedness among dental and pharmacy students for providing LGBTQ+ specific care.

**Methodology:**

This study is a retrospective observational study which used a novel interprofessional model of three different LGBTQ+ focused educational interventions within a group of dental and pharmacy students. The study used pre- and post-surveys, Assessment of Interprofessional Team Collaboration Scale (AITCS-II) and the Team Observed Structured Clinical Encounter (TOSCE) evaluations to assess the effectiveness of the interventions. Descriptive statistics, Fisher’s exact test, Wilcoxon signed-rank test, Welch test, Kruskal-Wallis Test, and pairwise Wilcox Test were employed to analyze quantitative data while qualitative insights were gathered from evaluator comments and student feedback.

**Results:**

The study evaluated cultural competence among 154 dental and pharmacy students revealing improved cultural humility post-intervention, particularly for dental students although not statistically significant (p>0.05). Students participating in multiple interventions had higher mean scores, but the differences were not significant (p>0.05). Significant differences were found among interprofessional teams of students in the domains of roles and responsibilities (p = 0.039) and patient centered approach (p = 0.039). No significant differences were found in individual scores participation in the teams (p = 0.018). Students also provided positive feedback on the program’s impact on their understanding of LGBTQ+ health issues and inclusive care.

**Conclusion:**

This program was a novel intervention aimed at improving cultural competence for health professional students in an interprofessional environment Further research in the direction can be useful in creating replicable programs.

## Background

Although acceptance of the Lesbian, Gay, Bisexual, Transgender, Queer or Questioning and others (LGBTQ+) population has increased in recent years, health disparities for LGBTQ+ individuals continue to exist [1, 2]. Disproportionate access to healthcare services among this population can be partially attributed to the lack of cultural competence among healthcare providers.

Lack of cultural competence has been found in the literature to be a significant barrier in providing appropriate gender affirming care [3–5]. To create a clinically and culturally responsive workforce, national organizations like the Fenway Guide to Lesbian, Gay, Bisexual, and Transgender Health have developed protocols to direct healthcare organizations in this regard [6]. As per the Health Quality Index report, only 36% of institutions that pursued organizational accreditation met the standards to be “LGBTQ + Healthcare Equality Leaders” [7]. Several LGBTQ+ advocacy organizations focus on education and training of healthcare providers. The Inaugural State of LGBTQ+ Health National Survey, administered by the National Coalition for LGBTQ+ Health (2022), highlighted the importance of integrating sexual health histories into routine clinical care and providing enhanced LGBTQ+ education, training, and support for healthcare personnel to deliver more personalized care [4]. The National Institute of Health, Sexual and Gender Minority Research Office also recommends mandatory inclusion of LGBTQ+ specific education for healthcare professionals to offer more individualized care and a more inclusive and welcoming environment to the LGBTQ+ population [8].

LGBTQ+ cultural competence training incorporates methodologies for healthcare providers and learners that include theory-driven, evidence-based, interprofessional, and multimodal approaches (1). More specifically, cultural competence training in health professions institutions underscore the importance of creating inclusive curricula and learning environments for the future healthcare workforce [9–12]. Within the last five years, there has been an increased emphasis on four main constructs of LGBTQ+-focused cultural competency training programs among healthcare professionals: knowledge, skills, behavior, and attitude [1, 13]. In some regions, such as Washington, DC, LGBTQ+ competence training is even required by law for renewals of healthcare providers’ licenses [14].

While there have been sporadic efforts to enhance LGBTQ+-specific education in healthcare curricula, there remains a gap in literature exploring the integration of an interprofessional perspective with LGBTQ+ instruction. Literature notes dental students report little interest in formal LGBTQ+ health education which may be attributable to the minimal exposure dental students receive as part of their training [14]. Dental students also report inadequate resources and support, as well as insufficient LGBTQ+ specific information at dental schools [15]. However, implementing LGBTQ+ competency training in dental education has been shown to improve implicit and explicit biases among future oral health care providers. Salter et al., focused on clinical cultural competency training for dental students using the Lesbian, Gay, Bisexual, Transgender Development of Clinical Skills Scale (LGBT-DOCSS), and found an increased feeling of clinical preparedness in treating LGBTQ+ patients, decreased bias toward LGBTQ+ patients, and increased knowledge of health disparities in the LGBTQ+ population after students were able to complete the training [16]. Another systematic review focused on training in dental, medical and nursing residents showed bias-focused educational interventions were effective at increasing knowledge; experiential learning interventions were effective at increasing comfort levels; and intergroup contact was effective at promoting more tolerant attitudes toward LGBTQ patients [9].

This study used a novel interprofessional model of three different LGBTQ+ focused educational interventions within a group of dental and pharmacy students. Therefore, the aim of this study was to evaluate the impact of the model in improving cultural competence and clinical preparedness among dental and pharmacy students for providing LGBTQ+ specific care.

## Methodology

### Study design

To address the aim of this retrospective observational study, a combination of three different interventions were employed: Student focused Project Extension for Community Healthcare Outcomes (ECHO) series; experiential learning opportunities at a gender health clinic; and interprofessional objective structured clinical examination (iOSCE). These interventions were offered as part of an elective course for dental students and included in part, as a component of a required IPE-based course for pharmacy students. The pharmacy students were required to participate in the ECHO series and were given the option of whether to join the other two interventions. The three interventions were employed between July 1st, 2023- April 30^th^, 2024.

The study protocol (#20142) was approved as an expedited review by the Institutional Review Board at Indiana University School of Dentistry (IUSD).

### Participant information and informed consent

The study included students from the Doctor of Dental Surgery (DDS) Program at Indiana University School of Dentistry (IUSD) and Doctor of Pharmacy (Pharm D) Program at Purdue University College of Pharmacy (PUCOP). A total of 158 (n = 23; 14.5%) dental and (n = 135; 85.4%) pharmacy students were recruited. 155 total (n = 20) dental and (n = 135) pharmacy students participated in the study. This sample was drawn from a total of 400 students enrolled in the DDS program at IUSD and included students from all four years of training. All students (n = 135) enrolled in the second year of the PharmD program at PUCOP participated. Before the study commenced, participants received an information sheet detailing the components of the study. Dental students were informed of the voluntary nature of the study, its purpose, and the intended use of collected data. Pharmacy students were informed of the ECHO series being part of their required course expectations, other information regarding the voluntary nature of the other components of the study was then shared. Consent for participation in any voluntary component of the study was received electronically. Dental students received credits for completing the study, whereas the ECHO series was included as a required course component. Pharmacy students could volunteer for the other components of the study but were not provided with any credit or other compensation for their participation.

### Data collection

Quantitative data were collected using pre- and post-ECHO surveys, as well as the Assessment of Interprofessional Team Collaboration Scale (AITCS-II Student) and the Team Observed Structured Clinical Encounter (TOSCE) measurements. Qualitative data from TOSCE evaluator comments and student feedback were also gathered to provide deeper insights into the effectiveness of the interventions. This design was chosen to capture both measurable outcomes and detailed participant experiences, ensuring a robust and comprehensive assessment of the program’s impact.

### Study interventions

#### Project ECHO

Project ECHO is an educational model designed to discuss health- and medical-related topics among a group of healthcare specialists, practitioners, and professionals to increase knowledge and provide information for standardized care practices via teleconferencing technology [17]. For this intervention, four ECHO sessions of 90 minutes (about 1 and a half hours) in length were delivered in collaboration with the Indiana University Indianapolis ECHO Center and a community partner organization specializing in education and training to promote LGBTQ+ inclusion. Two ECHO sessions each were offered virtually via Zoom in Fall 2023 and Spring 2024 for a total of four sessions. The dates for the ECHO sessions were (in the format DD/MM/YYYY): 29/09/2023; 17/11/2023; 20/02/2024 and 26/03/2024. The topics for the ECHO sessions were as follows: 1) Social identity, determinants of health and minority stress; 2) Emotional intelligence, language, and interrupting bias; 3) LGBTQ+ community and bias; 4) Trauma informed care, conflict, and hard conversations. Each session was attended by both pharmacy and dental students and began with a case study on a self-identified LGBTQ+ patient, with no personally identifiable information. Following the case study presentation, a facilitated case discussion was led by experts from the community partner organization. Additionally, a presentation was shared that expanded on key considerations for patient care, including information related to their gender identity, clinical concerns relevant to both dental and pharmacy students, social determinants of health considerations, and other care considerations relevant to the LGBTQ+ community. Experts from the community partner organization led an interactive discussion that expanded upon the topics noted above and included information relevant to the pharmacologic and dental care of LGBTQ+ patients. Participants were able to interact with the presenters and moderators by using the Zoom chat features, speaking during the meeting, or accessing the Mentimeter platform to submit anonymous questions and responses to polls.

#### Clinical experiences

The clinical experiences for dental students were completed during the Spring 2024 academic semester at a local Gender Health Clinic in Indianapolis, IN. Due to scheduling and logistic considerations, pharmacy students were unable to participate in the clinical experiences. The dental students participated in one, four-hour session, where interprofessional clinical supervisors provided a comprehensive educational approach designed to enhance the students’ understanding and skills in providing care to LGBTQ+ patients. Prior to attending the clinic, students were provided with optional reading resources addressing critical issues faced by the LGBTQ+ community in accessing dental care. The materials covered topics related to barriers to care and specific dental concerns and expanded upon topics covered during the ECHO sessions. Dental students’ utilization of the reading materials was not tracked for this study.

#### Interprofessional Observed Structured Clinical Examination (iOSCE)

During the Spring 2024 academic semester, students participated in an hour long iOSCE session, that was comprised of three stations via Zoom. A short break after each iOSCE station allowed the students time to complete the TOSCE survey before participating in a faculty-led debrief session. The sessions were designed and informed by the lived experiences of the LGBTQ+ simulated patients and providers. In stations one and two, students observed and assessed a scenario involving a simulated patient and simulated provider performing a patient intake and evaluated by the TOSCE form. In station three, students worked as a team to perform a patient intake on one of the simulated patients. The students were randomized into three interprofessional teams and attended one iOSCE session. Each session was facilitated by three simulated patients and/or providers, two moderators, and three evaluators. Evaluators assessed the interactions, focusing on the competencies outlined in the TOSCE forms. The evaluators met prior to the sessions to create inter-rater reliability with the TOSCE and review plans. During the debrief, students and simulated patients/providers engaged in interactive discussions to share perspectives, thoughts, and feelings about the stations. During the iOSCE interventions, anonymity of participants and evaluators was maintained. The names of the students and the evaluators were not revealed during the virtual sessions. The evaluators watched the session and were not observed on video. The evaluators identified the students by T-shirt color to score the TOSCE. An additional study personnel who did not participate in the evaluations collected all the TOSCE scores and matched them with student IDs, thereby maintaining anonymity.

### Instruments

#### Pre and post ECHO surveys

Pre and post-surveys were disseminated before the first ECHO session in the Fall 2023 semester and after the final ECHO session in the Spring 2024 semester (Appendix I). The surveys were adapted from a validated questionnaire previously utilized in similar research [13, 18–22], ensuring the reliability and relevance of the measures used. The survey items were designed to assess current educational space and curriculum, attitudes towards ongoing health equity education, comfort discussing sexual health with patients, perceptions of LGBTQ+ training adequacy, and views on instructors’ competency in LGBTQ+ patient care. The present study only assesses specific questions from the surveys as it aims to assess the overall intervention’s impact on items that specifically relate to domains related to curriculum inclusion, cultural competence and clinical preparedness. Responses were collected using a 6-point Likert scale ranging from "Strongly agree" to "Strongly disagree”.

#### AITCS II student

The AITCS II Student (Appendix II) is a tool used to measure the effectiveness of interprofessional teamwork within a healthcare setting [23]. The AITCS-II (Student) conceptualizes the essential characteristics of interprofessional collaboration as cooperation, partnership, team working, and coordination via 16 questions. Responses were self-reported by the students using a 5-point Likert scale ranging from 1–5: 1 for "Never," 2 for "Rarely," 3 for "Occasionally," 4 for "Most of the time," and 5 for "Always." The students were asked to complete it after all the above interventions. For this study, the use of the AITCS-II (Student) provided a reliable measurement to measure interprofessional collaboration during student team-based learning activities (i.e., the Gender Health Clinic and the iOSCE).

#### TOSCE

The TOSCE (See Appendix III) was used as the modified McMaster-Ottawa Rating Scale for assessing team and individual performance during the iOSCEs to evaluate simulated patient and provider interactions across six competencies: Communication, Collaboration, Roles and Responsibilities, Collaborative Patient-Family Centered Approach, Conflict Management/Resolution, and Team Functioning [24]. Similar to the AITCS-II (Student), the TOSCE was utilized to measure the students’ interprofessional teamwork. The team also received an overall global rating for the interaction. Students received rating scores of 1 = Below Expected; 2 = At Expected; and 3 = Above Expected for each competency. In addition to the TOSCE rating scores, evaluators included written comments on the team interactions with the simulated provider during station 3.

All surveys were administered using Qualtrics software to ensure secure data handling and participant anonymity. Participants were asked to use their unique identifier for all the surveys to maintain continuity without compromising their privacy.

### Data analysis

Descriptive statistics were generated for pre- and post-assessment of ECHO sessions from dental and pharmacy students, using median, minimum, and maximum values for continuous variables, and frequencies and percentages for categorical variables. Fisher’s exact test was applied to compare pre- and post-intervention responses, generating p-values for statistical significance. Combined data from dental and pharmacy students were recoded for consistency, and summarized using frequencies and proportions, with p-values calculated for comparisons between programs.

Data from the AITCS II Student tools were analyzed using the Wilcoxon signed-rank test since the data was not normally distributed. Since the sample sizes are unequal and the variances are not assumed to be equal, the Welch test was computed. The students were compared based on the activity participation and specific program. Mean scores for each AITCS-II (Student) survey item in the categories of Cooperation, Partnership, Teamwork, and Coordination were computed. Descriptive statistics were calculated; quantitative assessment of interprofessional collaboration as part of gender health experiences and iOSCEs were also calculated.

Descriptive statistics were compiled for the TOSCE evaluations including median and interquartile range on each of the seven evaluation categories. Team scores were compared using the Kruskal-Wallis Test due to the small sample size. If a significant difference was found in the competency score between the three groups, then a pairwise Wilcox Test with a Bonferroni p-value adjustment was performed to determine which groups differed from each other. Individual scores across the seven categories were compared using a Friedman Test. A summary of the qualitative data from the evaluators and students is also included. P-value was set at a threshold of 0.05. All numerical statistical analyses were conducted using R version 4.2.2 (R foundation for statistical computing, Vienna, Austria) [25].

## Results

Since portions of this project were voluntary, many students did not participate in all three interventions. For this article’s purpose, we included data relevant to each intervention.

“S1 Fig” shows the number of students participating in each of the interventions.

This study included 154 students who participated in the ECHO sessions. The participant population is described in “Table 1”. The majority were enrolled in the PharmD program (86%); the median age of the participants being 22 years, with an interquartile range from 21 to 32 years. Most participants identified as White (67%) and were not of a Hispanic (94%) origin. In terms of sexual orientation, the predominant group was straight (heterosexual) individuals, constituting 82% of the sample with 63% of the participants identifying as cisgender female.

**Table 1:** Demographic characteristics of the study participants.

Characteristic	N = 154^1^
*Program *	
Doctor of Dental Surgery (DDS)	21 (14%)
Doctor of Pharmacy (PharmD)	133 (86%)
*Age *	22.00 (21.00, 32.00)
*Sexual orientation *	
Bisexual	14 (9.1%)
Gay (men who love men)	4 (2.6%)
Lesbian (women who love women)	3 (1.9%)
Pansexual	1 (0.6%)
Queer	2 (1.3%)
Straight (heterosexual)	126 (82%)
Prefer not to answer	4 (2.6%)
*Pronouns *	
He/Him	48 (32%)
He/Him, They/Them	1 (0.7%)
She/Her	95 (62%)
She/They	1 (0.7%)
Prefer not to answer	7 (4.6%)
*Gender identity *	
Cisgender Female	97 (63%)
Cisgender Male	50 (32%)
Nonbinary/ Genderfluid / Genderqueer	1 (0.6%)
Decline to answer	6 (3.9%)
*Sex assigned at birth *	
Female	100 (65%)
Male	51 (33%)
Decline to answer	2 (1.3%)
*Race *	
American Indian or Alaska Native, White	1 (0.6%)
Asian	33 (21%)
Asian, Native Hawaiian or Other Pacific Islander, White	1 (0.6%)
Asian, White	3 (1.9%)
Black or African American	4 (2.6%)
Black or African American, White	2 (1.3%)
White	103 (67%)
Other	2 (1.3%)
Prefer not to answer	5 (3.2%)
*Ethnicity *	
Hispanic or Latino	5 (3.2%)
Not Hispanic or Latino	144 (94%)
Other	3 (1.9%)
Prefer not to answer	2 (1.3%)

^1^n (%); Median (Minimum, Maximum)

### LGBTQ+ inclusivity in climate and curriculum at the educational institution

“Table 2” presents different aspects of climate and curricular inclusivity at the educational institution. Most students (54%) agreed that their school had processes or mechanisms to address gender discrimination and to help patients address such discrimination. Less than half (40%) agreed the LGBTQ+ community was well represented and part of all conversations within the institution. When asked about the total hours dedicated to LGBTQ+ specific instruction, more than a third (38%) reported 3–5 hours; with lectures and presentations being the most common method of instruction (35%).

**Table 2:** LGBTQ+ inclusivity in climate and curriculum at the educational institution.

Indicate your level of agreement or provide information regarding the following statements related to LGBTQ+ inclusivity and education at your educational institution.	N = 154^1^
*The school has any process/mechanism to address gender discrimination in-house and to help patients address gender discrimination experienced in general *	
Agree	82 (54%)
Disagree	13 (8.5%)
Neutral/Undecided	58 (38%)
*LGBTQ+ community is represented well at each table and a part of all conversations in my institution *	
Agree	61 (40%)
Disagree	28 (18%)
Neutral/Undecided	64 (42%)
*My institution has an explicit mission (with objectives) on serving gender minority people *	
Agree	78 (51%)
Disagree	19 (12%)
Neutral/Undecided	56 (37%)
*My school has physical spaces and information/materials affirming to LGBTQ+ people *	
Agree	104 (68%)
Disagree	10 (6.5%)
Neutral/Undecided	39 (25%)
*I have received specific training or education on providing care to LGBTQ+ patients *	
Agree	98 (64%)
Disagree	15 (9.8%)
Neutral/Undecided	40 (26%)
*There are specific elective courses or modules dedicated to LGBTQ+ related topics at my educational institution *	
Agree	80 (53%)
Disagree	11 (7.2%)
Neutral/Undecided	61 (40%)
*How many total hours are dedicated to instruction on LGBTQ+ specific content in the curriculum*?* *	
Less than 1 hour	8 (6.7%)
1–2 hours	23 (19%)
3–5 hours	45 (38%)
6–10 hours	26 (22%)
More than 10 hours	18 (15%)
*Please specify how the LGBTQ+ specific content was covered in the curriculum*.* *	
Case studies and scenarios	31 (22%)
Educational videos or multimedia	3 (2.1%)
Guest speakers from the LGBTQ+ community	35 (24%)
Lectures and presentations	51 (35%)
Small group discussions	5 (3.5%)
Other	14 (9.7%)
I don’t know	5 (3.5%)

^1^n (%)

### Learning on topics in health equity

86% of dental participants agreed on the importance of learning about stigma before the ECHO sessions which increased to 94% after the intervention (p = 0.7). These results contrasted with pharmacy participants in that 75% of participants were interested in learning about stigma before the intervention, which decreased to 65% post-intervention (p = 0.061). Similarly, 86% of dental participants agreed on the importance of learning about cultural humility pre-intervention, increasing to 94% post-intervention (p = 0.7). This change was much smaller for the pharmacy participants, increasing from 75% to only 76% post intervention (p = 0.7). None of these changes were statistically significant “(Table 3)”.

**Table 3:** Learning on topics in health equity.

Indicate your level of agreement with the statement: ‘I believe it is important to learn more about the following topics in health equity’	Pre n (%) ^1^	Post n (%) ^2^	p-value^3^
**Doctor of Dental Surgery (DDS)**			
*Stigma *			0.7
Agree	18 (86%)	15 (94%)	
Disagree	2 (9.5%)	0 (0%)	
Neutral/Undecided	1 (4.8%)	1 (6.2%)	
*Cultural Humility *			0.7
Agree	18 (86%)	15 (94%)	
Disagree	2 (9.5%)	0 (0%)	
Neutral/Undecided	1 (4.8%)	1 (6.2%)	
**Doctor of Pharmacy (PharmD) **			
*Stigma *			0.061
Interested	58 (75%)	86 (65%)	
Neutral/Undecided	15 (19%)	26 (20%)	
Uninterested	4 (5.2%)	21 (16%)	
*Cultural Humility *			0.7
Interested	58 (75%)	101(76%)	
Neutral/Undecided	16 (21%)	23 (17%)	
Uninterested	3 (3.9%)	9 (6.8%)	

^1^ Total number of participants from DDS Program and PharmD Program in the Pre-intervention were n = 21 and n = 77, respectively

^2^ Total number of participants in the DDS Program and PharmD Program in the Post-intervention were n = 16 and n = 133, respectively

^3^Fisher’s exact test.

### Participation in gender health clinic experiential activities and iOSCEs

A total of 19 dental and 2 pharmacy students completed the AITCS-II (Student) survey which was distributed post participation in the gender health clinic experiences and the iOSCE. Seven (7) out of these 21 students completed both the above activities while 12 attended the gender health clinic only and 2 participated in the iOSCEs only.

Students participating in gender health clinic experiences (n = 12) generally had higher mean scores on all aspects than those that participated in iOSCE (n = 2), except for teamwork. However, these differences were not statistically significant. Similarly, students involved in both activities (n = 7) had higher mean scores than those in a single activity, but again, the differences were not statistically significant “(Table 4)”.

**Table 4:** Comparison of mean scores by activity.

Category	iOSCE^1^	Observational experiences at Gender Health Clinic^2^	p-value^2^	Both activities ^1^	Single activity ^1^	p-value ^2^
**Cooperation**	4.33	4.57	0.3	4.76	4.54	0.36
**Partnership**	4.00	4.42	0.45	4.43	4.36	0.71
**Teamwork**	4.6	4.56	0.67	4.89	4.57	0.27
**Coordination**	4.5	4.55	0.19	4.88	4.54	0.32

Mean ^1^; Welch t-test ^2^

Total number of students participated included 19 DDS and 2 PharmD students

Dental students (n = 19) perceived better cooperation, partnership, teamwork, and coordination within their teams compared to pharmacy students (n = 2). However, these differences were not statistically significant. Thus, the type of program did not significantly impact these aspects of team dynamics “(Table 5)”.

**Table 5:** Comparison by degree.

Category	Doctor of Dental Surgery (DDS) 1 N = 19	Doctor of Pharmacy (PharmD) 1 N = 2	Others ^1^	p-value^2^
**Cooperation**	4.68	4.33	4.0	0.25
**Partnership**	4.44	4.00	4.0	0.43
**Teamwork**	4.72	4.63	4.0	0.56
**Coordination**	4.68	4.50	4.42	0.18

Mean ^1^; Welch t-test ^2^

As per the results from TOSCE “(Table 6)” descriptive statistics indicated that one of the teams, (Team 3) performed the best out of the three teams, performing above expected in five of the six competencies and an above expected Global Rating. Overall, each team and member performed at or above expected in the Team Functioning competency. Kruskal-Wallis tests determined a significant difference in the Team scores for Roles and Responsibilities (p = 0.039) and Patient Centered Approach (p = 0.039). A moderate difference was found between Teams 1 and 3 in these two categories based on the pairwise Wilcox Test (p = 0.074).

**Table 6:** Median TOSCE scores by category.

Team	Communication^1^	Collaboration^1^	Roles & Responsibilities^1^	Patient Centered Approach^1^	Conflict Management^1^	Team Functioning^1^	Global Rating^1^
**1**	2.5 (2–3)	2.5 (2–3)	2	2	2 (2–2.5)	2 (2–2.25)	2 (2–2.25)
**2**	3 (2.5–3)	2 (2–2.5)	2 (2–2.5)	2 (0.5)	2	2 (1.5–2.5)	2 (2–2.5)
**3**	3	3	3	3	3 (2.5–3)	3	3

^1^Median (Quartile 1—Quartile 3)

Total number of students participated included 8 DDS and 2 PharmD students

No significant differences were found in individual scores by the Friedman Test, implying all students w ere performing at the same level and the educational content was received effectively by this group of participants (p = 0.18).

### Qualitative feedback

Evaluators provided comments on the team performance. Team 1 worked well together and used effective listening skills and a collaborative approach. Team members provided detailed introductions and focused on the patient’s concerns, with each member speaking with the patient on different treatment and care areas. Team 2 had a good discussion on roles, patient readiness, and medication use, but struggled with post-introduction to hand off the patient smoothly to each other. Team members handled the patient’s concerns and feelings well but could improve on collaborating and exploring the patient’s social determinants of health. Team 3 was very thorough and empathetic with effective handoffs, clear roles, and a strong rapport with the patient. All team members used the correct pronouns and supportive language consistently throughout the interaction.

Students also provided feedback during the debrief sessions. Students expressed their appreciation for observing two scenarios (i.e. iOSCE Stations 1 and 2, respectively) that differed greatly in tone and effectiveness from each other. In Station 1, the provider was disrespectful and made derogatory comments about the LGBTQ+ community without knowing that the patient identified as LGBTQ+. One student stated that “I am glad this scenario was part of the OSCE because it raised a lot of red flags… It was good to see a disrespectful interaction that way I knew if it ever happens in my future career, I know that I would insert myself into the conversation to put a stop to the harassment.” In Station 2, the provider performed a very thorough and trauma-informed intake on the patient and considered many aspects of care, treatment, and referrals. A student commented that “This is how every appointment should go, as it provides the patient with ease and a calming experience that was once a terrifying one.” Students also commented about the year-long intervention. The feedback was very positive with dental students expressing their desire for this course to be part of the overall curriculum rather than just an elective (pharmacy students participated in the ECHO series as part of a required course). One of the students stated:“I learned a great deal about local resources, and I feel much more aware of some of the barriers patients who are part of this community face when accessing/navigating healthcare settings.” Another student noted, “I definitely learned a lot from this especially about interdisciplinary care with the SP [simulated patient] and trauma-informed care.” Additionally, a student appreciated the presence of two different scenarios, stating, “I appreciate there being two different scenarios rather than just one. This provides us with the ability to compare the two, to see a great interaction with an uneasy interaction and how we should handle each patient with care and respect”. The qualitative feedback highlights the importance of integrating diverse, scenario-based learning experiences and reflects students’ strong desire for such training to be a core component of their education.

## Discussion

The study results align with broader trends in literature that indicate a growing but uneven commitment to LGBTQ+ inclusivity and education in educational institutions. While progress is being made, especially in providing training and affirming spaces, there remain significant gaps in representation, explicit mission alignment, and comprehensive educational offerings on LGBTQ+ issues.

The study results did indicate a majority of the respondents believed having received some training or instruction specific to LGBTQ+ content at their institution, which compares to research by Sharma et al. (2021), while many healthcare education programs include some level of LGBTQ+ related training, the depth and comprehensiveness of this training often vary [26]. Comprehensive training is crucial for improving the quality of care provided to LGBTQ+ patients [26]. Although there is no ideal way to train future oral health providers, at a minimum, complying with the standards of Commission on Dental Accreditation requires delivering content on the following topics: 1. Sexual orientation, gender identity, and LGBTQ+ terminology 2. Health needs and risks 3. The potential impact of LGBTQ+ related discrimination on health inequities 4. Provision of inclusive care [11]. Similarly, the Accreditation Council for Pharmacy Education (ACPE) Standards require pharmacy graduates be able to 1) describe how population-based care influences patient-centered care and the development of practice guidelines and evidenced-based practices; and 2) recognize social determinants of health to diminish health disparities and inequities in access to quality care [27].

The literature on LGBTQ+ experiences in professional education predominantly addresses the medical environment. Per a report published in 2014 by the Association of American Medical Colleges (AAMC) Advisory Committee on Sexual Orientation, Gender Identity, and Sex Development, 30 competencies within 8 domains to address the needs of LGBTQ+ patients in medical education were recommended [28]. These competencies are built within eight existing competency domains: 1. Patient Care 2. Knowledge for Practice 3. Practice-Based Learning and Improvement 4. Interpersonal and Communication Skills 5. Professionalism 6. Systems-Based Practice 7. Interprofessional Collaboration 8. Personal and Professional Development. The intervention, being one of the few addressing both dental and pharmacy education curricula with its multi-intervention approach, touched upon all the above-mentioned recommended domains. Per a recent systematic review, majority of studies exploring inclusion of LGBTQ+ specific content in medical curriculum involved preclinical interventions [29]. Our program offered both preclinical and clinical interventions in the form of ECHO sessions which focused on didactic topics and case discussions, followed by interactive observational experiences with patients from the LGBTQ+ community and the iOSCE sessions that included simulated patients and providers representing the LGBTQ+ community.

The ECHO survey results align with literature in highlighting that while lectures and presentations are the most common methods for teaching LGBTQ+ content, there is growing recognition of the value of more interactive and diverse teaching methods. The hours of instruction reported in the survey reflect a range that is consistent with findings from a recent systemic review by Yu et al. (2023), who advocate for more extensive and comprehensive training [12]. Interactive sessions and small group discussions have been also found to be particularly effective for engaging students and facilitating deeper understanding, although these methods were less commonly reported in the survey [30, 31]. While both programs showed shifts in attitudes toward learning more about stigma and cultural humility, the trends observed in the dental and pharmacy programs differed in magnitude and direction. This difference likely indicated a desire by dental students to continue to learn more about these topics as they progress in their education and training, but pharmacy students appeared to be content with where their knowledge was related to these topics at that stage in their education. ECHO survey results also showed most students agreed their school provides the spaces and infrastructure for gender affirming practices. Research by Keuroghlian et al. (2017) shows that affirming environments, including visible support materials, significantly enhance the well-being and academic success of LGBTQ+ students [32].

Our study also indicated improved interprofessional collaborative outcomes, especially for dental students. This aligns with study conducted by Prasad et al. (2023), that suggested that an interprofessional case-based learning approach for students across different healthcare professions significantly enhanced cultural competence to provide collaborative and standardized care for LGBTQ+ individuals [33]. Collaborative health care teams are found to be leading to increased quality of patient care, safety, and positive outcomes; reduced health care costs; and greater satisfaction among patients and providers [34, 35]. Students that participated in both clinical experiences and iOSCEs had better scores than those that participated in only one. While the differences were not statistically significant, the higher mean scores suggest that participating in multiple activities may enhance specific team dynamics and planning processes, likely due to increased exposure and interaction in diverse settings. The literature supports the importance of clinical rotations in building interprofessional team-based care skills [36].

Significant improvements in participants’ collaborative skills and knowledge related to LGBTQ+ health equity post cultural competence training was noted by a study by Leslie et al,. These findings complement our study where the educational model did improve the cultural competence for the participants, especially with respect to treating patients from the LGBTQ+ community [37]. Our multipronged approach aligned with studies like the one by Sekoni et al. (2017) which demonstrated significant improvements in knowledge, attitudes, and clinical skills related to LGBTQ+ health among healthcare students and professionals following targeted educational curricula and training, particularly emphasizing the effectiveness of experiential learning methods and the need for ongoing education to sustain these improvements [11]. While it is ideal for students to be able to get clinical experiences specific to the care of LGBTQ+ patients, getting students in these settings can be logistically difficult. If these experiences were not available, the results of this study show that directed education related to LGBTQ+ care can still lead to improvements.

Findings from the TOSCE align with existing literature on team-based clinical assessments, emphasizing the critical role of effective teamwork and communication in achieving superior performance outcomes [38]. The observation from study results (Table 6) indicates that Team 3, scored high (3) in all domains which aligns with the evaluators positive feedback about effective handoffs, clear roles, and a strong rapport with the patient. This resonates with studies highlighting the impact of cohesive team dynamics leading to better performance in clinical settings [39]. Team 1 displayed effective listening and teamwork skills which is consistent with their median TOSCE score of 2.5 in communication and collaboration categories. Though these scores were not the highest, the evaluators highlighted the team’s effective listening and collaborative skills. Additionally, the identification of individual performance discrepancies within the team highlights the significance of individual contributions within a team context, reflecting broader literature on teamwork and collaboration in healthcare [40]. For instance, in Team 1, even though communication and collaboration scores were moderately high (2.5), other categories were scored low (2) which could be due to inconsistencies in how each individual in the team contributed to the team’s performance. The significant differences in scores for patient-centered care across teams, underscore the importance of patient-centered approaches in contemporary healthcare delivery models, emphasizing the need for targeted interventions to optimize patient outcomes and satisfaction [41, 42]. Overall, the feedback from evaluators indicated that although the team members displayed empathy for the patients, there was still room for improvement in terms of cooperation and including patient’s social determinants of health in patient care. This is supported by literature suggesting that improving patient outcomes when addressing social determinants of health is essential with a collaborative approach in addition to efficient communication [43, 44].

The positive feedback about the year-long intervention from student participants supports the usefulness of such programs and serves as an example for educators to look for opportunities to include this type of focused education and training in their curricula. In a similar study by McCave et al. (2019) an interprofessional simulation training was implemented for 2 years, and 90% of the student participants felt the training prepared them better in the interprofessional competencies [45]. Future studies should evaluate long-term retention of LGBTQ+ cultural competence trainings across diverse student body at multiple healthcare institutions, measure its impact and its practical application in clinical settings [45]. Supporting our work, recent studies like the one by Bass et.al reaffirm that, better patient outcomes for LGBTQ+ patients are achieved when providers use inclusive language, understand specific healthcare risks, and offer tailored, knowledgeable care [46]. It’s important to note that our study, like several studies, measured the impact of the program by using pre and post surveys, however program effectiveness measurements without long-term evaluation is still fragmented [46, 47]. Further evaluation of the long-term program impact should be explored with future studies [46, 47]. Also, since this intervention was offered only once as an elective course, the impact of the educational interventions should be reviewed by offering such programs more frequently in the curriculum [46, 47].

### Strengths

Our program was interprofessional and looked at aspects of cultural humility and competency and followed a multi-interventional approach. This ensured evaluation of the impact of the program from more than one perspective. The study also used validated tools to measure interprofessional collaboration, which showed significant improvement post participation in the program. Student feedback was encouraging and highlighted their interest in continuing to learn.

### Weakness

This program was offered in different capacities for the learner groups (i.e. as an elective for dental students, and with both required and elective components for Pharmacy students), so it was difficult to get all students to participate in all the activities. Moreover, the tightly packed curriculum in dental and pharmacy courses allowed very little time to participate in elective activities. The study sample may have led to selection bias because of the voluntary nature of participation, for dental students especially so the students who may have signed up to participate, may already have an interest in the topic. As this training can be classified under cultural competency training, research indicates that individuals who are already sympathetic to other cultural populations are more likely to engage in voluntary cultural competency training. Therefore, some components of this training may not have reached the population of students who are in need of these trainings.

## Conclusion

This program was a novel intervention that aimed at improving cultural competence for health professional students in an interprofessional environment. Further research in the direction can be useful in creating replicable models. Health professions programs offer a unique opportunity to shape the future healthcare workforce. With them embracing diversity, equity and inclusion, specific to LGBTQ+ instruction, significant improvements in health equity for this vulnerable group can be expected.

## Supporting information

S1 Fig Number of students participating in each intervention. (TIF)

S1 Data Pre_InterventionSurvey_Dental. (XLSX)

S2 Data Post_InterventionSurvey_Dental. (XLSX)

S3 Data Pre_InterventionSurvey_Pharmacy. (XLSX)

S4 Data Post_InterventionSurvey_Pharmacy. (XLSX)

S5 Data iOSCE_data. (XLSX)

S6 Data iOSCE_COMMENTS. (DOCX)

S7 Data AITCS. (CSV)
