# Supplementary figures and images for: An interprofessional model to improve LGBTQ+ specific cultural competence in dental and pharmacy students
Source: PLoS One. 2025 Jan 9;20(1):e0313492. doi: 10.1371/journal.pone.0313492 (PMC11717247; doi:10.1371/journal.pone.0313492)

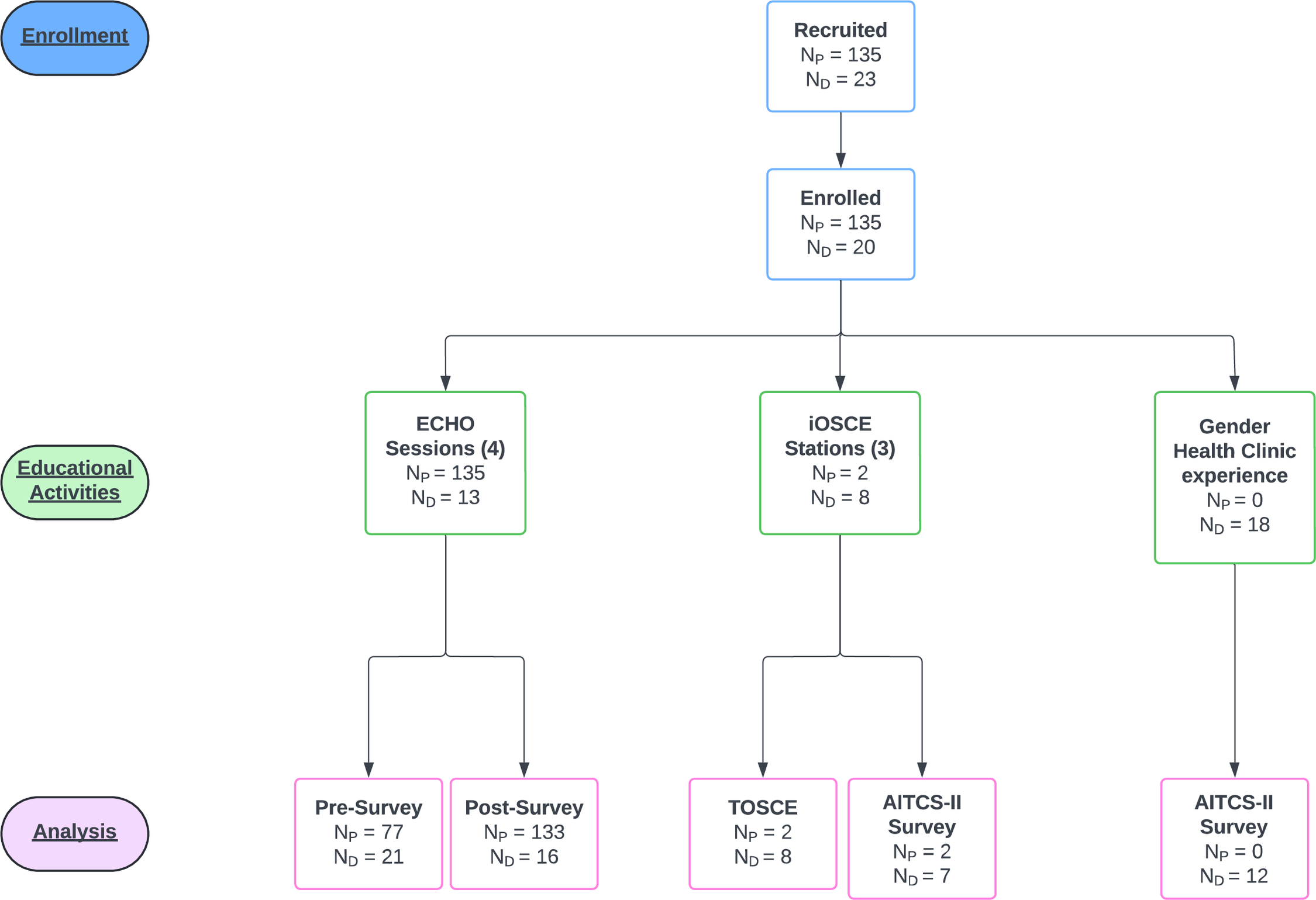

Supplement: S1 Fig — (TIF) [file pone.0313492.s001.tif]
