# Supplementary material for: An interprofessional model to improve LGBTQ+ specific cultural competence in dental and pharmacy students
Source: PLoS One. 2025 Jan 9;20(1):e0313492. doi: 10.1371/journal.pone.0313492 (PMC11717247; doi:10.1371/journal.pone.0313492)
Supplement: S6 Data — (DOCX) [file pone.0313492.s007.docx]

**EVALUATOR COMMENTS:**

Session 1 (April 16th):

Intake 1: Answered patient questions. Use of word “sucks”; trying to distract with dog conversation.

Comments that are present next to the table:

-No pronouns stated, provided inappropriate family questions. Short, quick and not thorough. Offer returns. Patient decline reviews reasons.? Of reactions. Anxiety. Make sure fine ID-address rationale for asking questions. Mentions about being “gay” and not having HPV reviewed procedure.

-Clinical Greetings: Apologized

-Clinical Introduction: name, title, apologized

-Clinical Receptivity: reports are staffed

-Patient Alliance Building: a little off-putting

-Procedure explanation: brief but ok

Intake 2: Asked about concerns of teeth; good listener-nodding, “great smile”; reassurance to assist as needed; very through explanation of are interventions; recognizes anxiety & ask if okay.
Provides sunglasses and ear plugs/music; offered “taking break”.

Comments that are present next to the table:

-Very thorough compassion care. Asked stable housing, safe home, others phone #. Concern about any trauma to mouth, face, jaw. Reports will send a referral for the same visit. Rationale for perineal (not sure of this word) questions-extra care for LGBTQ+ status.

-Clinical Greeting: nice greeting

-Clinical Introduction: good name; title; pronouns

-Clinical Intake thoroughness: reviewed procedure.

Intake 3 (17AB54; Black): Good listening behavior. Addressed a specific area of care (plan and procedure)

Comments that are present next to the table:

-Questions for dental/oral health medical care.

Questions are feeling coming to dentists: -any nervousness; -bad experiences reassures patient during appointment and mitigation. Willing to assist patients throughout examination.

-Expressed support/ compassion team.

-Clinical Greeting: explained visit

-Clinician introduction: name, title, pronouns.

-Clinical Intake thoroughness: explains procedure

-Clinician’s Receptivity: compassionate care

-Trauma-Informed Communication: dental trauma treatment.

Intake 1: Encountered based on getting the procedure done process focused not person focused. Rushed, not ready to ask or listen to the person. Felt like they just wanted to get it done.

Comments that are present next to the table:

-Apologizes/ only one here/ challenges come back.

-Clinical thoroughness: I hear you. Let me check system. I know this is silly talking about suicide. Shocked you HPV (not sure what is written)

-Clinical introduction: only introduced themselves. One sided.

-Clinical intake thoroughness: Did not ask about person

-Clinical receptivity: Auditioned fear but superficial.

-Patient Alliance Building: Dogs when patient doesn’t have any.

-Informed Consent: Assumed

-Procedure Explanation: Not really

-Collaboration between patient and children: absent

-Appropriate screening for SDOH relevant to patient: Not done

-Adequate Resourcing: None

Relevant Referrals: None

Intake 2: Person-centered approach. Took time to learn about the person and tailor the encounter to their unique situation

Comments that are present next to the table:

-Clinical Greeting: I will be with you. Pronouns asked.

-Patient Alliance Building: Listening

-Trauma-Informed Communication: Injuries in Past. Extra time allocated. Is that stable. Falls.

-Informed consent: asked about body injuries + comfort

-Procedure explanation: explained step by step

-Appropriate screening for SDOH relevant to patient: Asked about HIV and asked about viral load.

-Relevant referrals: Happy to send a referral. Help get you to the services you need Julian center.

Intake 3 (05FI82; Green): Group did a collaborative assessment. Demonstrated effective listening. Did not ask specific questions. Explained procedure well

Intake 1: Participant was very distracted

Intake 2: This scenario was very well done, thorough. Great example for students

Intake 3 (16PE63 Grey

29KU56 Navy): Overall worked well as a team. Difficult to use this tool with so many participants when each had a role

Session 2 (April 22nd):

Intake 1: Rushed, lack of empathy. No informed consent. Not addressing patient’s concern or anxiety.

Intake 2: Takes time to address patient’s concerns. Provides information about the procedure and asks for consent. Trauma informed care and communication. Empathy and patient centered communication

Intake 3 (07HA64; Maroon): The team lacked collaboration in terms of IPE. The conversation did not specifically address trauma informed care

Intake 1: None

Intake 2: None

Intake 3 (30ZI70; Tan/Brown): Huddle: prepare patient; discuss ER, introduce selves; make sure patient comfort levels; assess pain level and dental anxiety; readiness for procedure; ask last time with work completed; prepare pt for care; if taken med for pain, if in pain; silence was okay

M-listener appropriately; appropriate behaviors-nodding, smiling; able to discuss options for financial services; good communication

Intake 1: Good apology for having to wait, but it didn’t sound genuine
-Good job confirming name and address - correct patient
-Good perspective on why introductory questions are being asked
-Good exploration of medication coverage, to help understand what coverage could look like
-Good recap on information and confirmation of services to be performed
-Did not ask patient about their preferred pronouns, names, etc.
-Inappropriate comment about HPV vaccine - shocked they don’t have it already unless they’re gay
-Good job asking for any questions or other concerns before getting started and addressing these concerns as best as possible
-Exploration of work and having blue hair is judgmental – seemed like this was used to make small talk for a patient that was nervous, but not the appropriate way to do this
-Need to wrap up with offer to answer any final questions
-Felt rushed, annoyed to have to do shots in the midst of rest of their job
-Acknowledged that patient is fearful of needles and a lot of people are, but didn’t address their concern - telling me others are concerned doesn’t necessarily alleviate my own concern.

Intake 2: Good introduction with name, pronouns, roles, and what to expect during the session
-Good asking patient about preferred name and pronouns
-Good flexibility about address issue and confirmation of whether it will be stable housing
-Good confirmation of demographic information and access/availability of contact information
-Above normal role - offer a referral for insurance navigator, or someone else to help with payment, other services for HIV care
-Tone of conversation was reassuring, understanding, appeared genuinely interested in helping
-Good exploration of why care services might have been interrupted over the year and goals for this current encounter
-Good re-explanation of the session in more detail as it was getting started, including procedures, who will be there, what will be happening, etc.
-Good wrap up to the session with a chance for the patient to provide any final concerns or questions
-Good job bringing patient to a private space to address more sensitive questions, and letting the patient know these questions will be coming and why they’re being asked
-Made patient feel very comfortable to share their concerns, even on very personal matters - there was never just judgement for information the patient shared
-Great job offering ways to help patient mitigate stress or anxiety associated with the dental procedure, and the offer for how to let the dental professional know if they need to take more time or a break

Intake 3 (06we67; Black):

Huddle
-Good offer of how to approach the conversation among multiple team members
-Good discussion of making sure to introduce each team member
-Great discussion to determine patient’s readiness for a procedure, concerns in this setting, when they last had dental work done, etc
-Good consideration of roles, Most students thinking about prior care and some students considering what medications patient has taken

Interview
-Good job starting with introductions, professions, and pronouns - among all team members
-Look for chances of handoff in your interview, after one person asks some questions, can pass it onto another member to see if there’s more things to explore
-Good collaboration with the patient and asking for their goals about whether they have a preference for keeping the tooth
-Team struggled with a plan after initial introductions and explanation of what to expect during the procedure - look for the chance to transition into the rest of the interview - you can ask the patient if they have any further concerns they’d like to address before going into the procedure - relied on the patient to be more forthcoming with this information
-Look for more chances to explore SDOH and/or other risk factors for the dental concerns they have - this was largely provided by the patient without specific prompting from the group
-When identifying there is no option for dental insurance through the patient’s employer - look for the chance to make a referral to someone who can help with this (but there was a mentioned of considering alternate payment options).

Session 3 (April 22nd):

Intake 1: Rushed intake. Patient concerns not addressed

Intake 2: Great patient centered conversation. Takes into account patient’s concerns

Intake 3 (10NO66; Black): Very thorough, collaborative and empathetic!!

Intake 1: None

Intake 2: None

Intake 3 (08SX73; Blue): Huddle: good questions for medical conditions related to procedure; what's medication list; type of asthma; have inhaler with them; more history about tooth, any pain, what's causing pain; determining cause of tooth situation-for patient to answer open and honestly; discuss insurance/ability to pay, use proper pronouns used; also review other health and conditions.

Student J: good informed comments in huddle; cooperative with team; moved quickly from when patient said "I could be better"; good questions about dental care, falls injury to head or face; safety at home; explained options for intervention; excellent communicator.

Intake 1: Good brief introduction and apology for having to wait, but seemed very rushed throughout the encounter
-Good brief perspective on why certain questions were being asked
-Seemed to deflect any additional work or processes that needed to be done (i.e. address and insurance updates, etc.)
-Good review of services to be provided today and review of vaccines scheduled to be given
Inappropriate comment about HPV vaccine - shocked they don’t have it already unless they’re gay
-Largely blew off the patient’s concerns related to anxiousness with shots, especially saying fainting is no big deal and they’d catch them if they fall
-Good that the provider offered apologies throughout, but they didn’t seem genuine
-Overall, there were a lot of chances to better support the patient throughout this encounter and addressing their fears related to the shots, instead of just quickly moving past these. That being said, the interaction portrayed is representative of a many similar encounters in practice

Intake 2: Good introduction with name, pronouns, roles, and what to expect during the session
-Good asking patient about preferred name and pronouns
-Good confirmation of demographic information and access/availability to this contact information
-Good brief perspective on why certain questions were being asked - largely as part of general intake questions all people are asked at this site
-Tone of conversation was reassuring, understanding, appeared genuinely interested in helping
-Above normal role - offer a referral for insurance navigator, or someone else to help with payment, other services for HIV care, etc.
-Good job including the patient in the conversation and learning more from them about oral health history, goals of the current encounter, etc
-Supporting self-efficacy was displayed well, with the provider supporting the patient’s goal of having good oral health moving forward
-Good explanation of the dental session in more detail as it was getting started, including procedures, who will be there, what will be happening, how long it should take, etc.
-Good job bringing patient to a private space to address more sensitive questions, and letting the patient know these questions will be coming and why they’re being asked
-Made patient feel very comfortable to share their concerns, even on very personal matters - there was never any judgement about information the patient shared

Intake 3 (**23EA62; Dark Blue**):

Huddle:
-Good introduction of team roles at start of the huddle, and initial focus on addressing patient’s primary concerns and ability to communicate these in the midst of their current dental issues
-Good consideration of exploring the patient’s dental history, not just with the current tooth issue, but also in the past (and considering if there was something like a domestic issue that could be a cause for the dental issues)
-Overall, the team came together well to identify their list of goals and priorities for what to accomplish during the current dental procedure
-Great job acknowledging the use of the proper pronouns with the patient, whether they have reliable transportation for follow up care, etc.
-Excellent example of team roles where one person was unfamiliar with something that was specific to another profession, asking for clarification on this, and willingness of the professional in this space to provide the explanation (another good example related to some unfamiliarity around the impact of the patient’s thyroid disorder)
-The conversation during the huddle was outstanding, this included the discussion of how you want to balance your own conversation as team!

Interview:
-Good introduction of team members and roles on the team, and asking patient of their preferred pronouns
-Excellent job asking the patient about their comfort with talking through issues given the presence of their current dental issues
-Good job clarifying what medications the patient is taking, can ensure whether these will impact the upcoming dental procedure
-The tone of the interview was very good, in that there was no judgment related to any information shared with the patient, the patient felt supported with the language used by team members
-Roles and responsibilities was great in the interview and picked up exactly where you would want it to based on the conversations had during team huddle - this included offering for the patient to get other referrals to people who could best help support their concerns.
-Great handoffs among team members! Each of them asked their set of questions, then let the patient know they were going to hand the conversation off to their colleague to explore some other things in more detail
-Roles identified during the team huddle were fulfilled perfectly during the interview…excellent job!
-Great wrap up to the session to round out the conversation, answer any lingering questions, reviewing relevant information discussed during the encounter, etc.

MODIFIED MCMASTER-OTTAWA RATING SCALE: Team Rating scale:

Session 1 (April 16th):

29KU56; Navy: Definitely took the lead; could work on hands off to other providers

16PE63; Grey: Found ways to hand off to peer. Introduced that "Would cover next steps

17AB54; Black**:** Appropriate intro, supportive environment, demonstrate good listening

behavior, nice smile, friendly approach, provides pear and expectation, location distraction.

05FI82; Green: Took the lead for the huddle and started the planning process and the encounter. Nice handover to colleagues; Direction to resources related to payment. Also asked Actor 2 about their thoughts on the suggestions. Very person-centered approach.

Team instrument1.1: Spend time huddling to plan on responsibilities, so that team can contribute fully. Functioned well with each other given online component. Correct back up behavior when zoom failed.

Team instrument1.2: Students collaborated well in the huddle. We are here to help you use in the introductions. "How you feel about coming to the dentist". The question was well placed and drew in the personal perspectives. Use of a lot of talk about together we can work it out. Student M described the process would be good to share strategies to help Actor 2 feel safe.

Team instrument1.3: Huddle: reviewed intro with name, pronouns, title, ask medications. Q-GERD secondary erosion. How to start decided; ask about clarifying questions. Explain rationale of ER appointment.

Encounter: supportive intro; asked pronouns; CC to ER reinforced no embarrassment or judgement reassuring patient pain and teeth trauma.; worked through distraction of “M’s” mic.

Team functioning: good summary

Global rating scale: no LGBTQ+ specifics noted.

Actor1 & Actor2 1.1: Difficult to score team questions with other team members present, however teamwork with patient was great. Very thorough and empathetic of patient’s stated and unstated needs. Developed rapport well with patient

Actor1 & Actor2 1.2: None

Actor1 & Actor2 1.3: Collaborated and connected with the patient. Took time to explain all steps and asked relevant and important questions that were specific and tailored to Actor 1 situation. Person centered action clearly demonstrated

Actor3 & Actor2 1.1: Participant make comment about being gay and relationship that is HPV, does not seem empathetic to patient

Actor3 & Actor2 1.2: None

Actor3 & Actor2 1.3: Rushed and doesn't learn anything useful about the person. Just administer injection focus

Session 2 (April 22nd):

07HA64; Maroon: None

30ZI70; Tan/Brown: None

06we67; Black: Huddle
- Good recommendation of place to start the conversation, and identifying some potential goals of the procedure and risks associated with the procedure(s) that might need to be performed

Interview
- Good consideration of how patient might be feeling at the start and asking about prior dental care
- Look for the chance to reflect the concerns and circumstances the patent communicated to you, to make sure you heard them correctly
- Good job considering the patient’s pain and anxiety, and your goal of trying to mitigate these
- Good explanation of why the patient is experiencing the pain or sensations they have in their broken tooth

Team instrument 2.1: None

Team instrument 2.2: None

Team instrument 2.3: None

Actor3 & Actor2 2.1: None

Actor3 & Actor2 2.2: None

Actor3 & Actor2 2.3: None

Actor2 & Actor3 2.1: None

Actor2 & Actor3 2.2: None

Actor2 & Actor3 2.3: None

Session 3 (April 22nd):

10NO66; Black: None

08SX73; Blue: None

23EA62; Dark Blue: Huddle
- Good consideration of patient’s medications, PMH, and insurance information - and how these could have an impact on the current dental services that need to be performed

Interview
- Good job asking if it was ok to ask certain questions to the patient regarding the current injury, then diving in to investigate this in more detail (e.g. level of pain, how it happened, when it happened, things making it better or worse, etc.)
- Great job confirming consent to treat, then a perfect handoff from there to the person who was going to talk about treatment options

Team instrument 3.1: None

Team instrument 3.2: None

Team instrument 3.3: None

Actor2 & Actor1 3.1: None

Actor2 & Actor1 3.2: None

Actor2 & Actor1 3.3: None

Actor1 & Actor2 3.1: None

Actor1 & Actor2 3.2: None

Actor1 & Actor2 3.3: None

**STUDENT COMMENTS:**

April 16th:

**Station 1**

05FI82; Green: Overall, not very respectful of different perspectives
Spoke to the patient as if they were an inconvenience
Did not give the patient much time to speak
Didn’t really demonstrate active listening or a kind approach to the patient’s discomfort

29KU56; Navy: Comments: I felt they did ok explaining the parts of the actual procedure, but provider made comments that likely made pt feel uncomfortable and only really addressed exactly why the pt was there instead of diving a little deeper to make sure pt was overall in good health/safe.

17AB54; Black: The provider read the patient’s body language and asked about their anxiety
Asked if they feel safe at home, and stated they ask everyone. Dismissed Actor 3 when she brought up her spouse.

16PE63; Grey: The participant was very cold and said microaggression multiple times towards the patient including "only gay people get HPV" which was totally unprofessional and hateful. The participant lacked empathy for the patient's anxiety and brushed off the patient multiple times.

**Station 2**

05FI82; Green: Demonstrated really good listening skills by showing the patient that they took time to offer solutions or at least some feedback about the patients concerns
Made sure to clearly map out the procedures and what the patient should expect
Took into account all of the possible reasons that a patient may be uncomfortable and addressed them appropriately

29KU56; Navy: very effective communication. Provider reassured/ comforted pt several times.
Could have asked some questions in a more private setting to begin, but glad provider confirmed questionable answer when they did finally move to an exam room. Loved that they offered referrals for other issues pt may be experiencing aside from dental treatment.

17AB54; Black:

-Trauma informed care
-Asked if the number on file was safe to call and share private info
-Waited until they were in the room to ask more sensitive info questions
-Explained the plan for the day, who was going to be in the room and asked if any known triggers before starting the procedure
-Offered comfort items like headphones, sunglasses
-Not sure if touching the dentist is a good idea. Even with our non-dominant hand sometimes we’re holding the mirror, or a retraction instrument and it could cause injury to the patient.
-Need for referrals was identified

16PE63; Grey: I have had some experiences like this interaction before. I like that the dental
professional was very empathetic and listened to the patient. I really liked the introduction with pronouns (unlike the last station), and the reassurance from the dental professional. I would say I disagree with the touching to signal distress- that can be very dangerous in our profession but other than that I thought this station was way better than the last

April 22nd:

**Station 1**

10NO66; Black: -HPV comment “most people our age, already have HPV”
-did not address patient fainting fear very well, dismissed fears
-did not use professional language throughout interaction
-did actively apologize for long wait time

30ZI70; Brown: “most people have it unless you’re gay” is very derogatory

23EA62; Dark blue: The provider had a few issues with communication and collaboration. The provider made a comment “that is surprising, most of the people our age is HIV? negative in the Queer community” or something similar to that that I thought was not very nice to the patient and caught them off guard. The provider also made a comment that if they fainted it was, “no big deal and I’ll catch you if I have to,” which also could have been phrased better. They handled the conflict with the patient wanting to leave before 10 minutes very well. The provider could’ve spent more time building rapport and making the patient comfortable at the beginning, even though they were late & understaffed.

06we67; White: I am glad this scenario was part of the OSCE because it raised a lot of red flags in the comments that the doctor was saying about the patient’s hair, job, and HPV vaccine. It was good to see a disrespectful interaction that way I knew if it ever happens in my future career, I know that I would insert myself into the conversation to put a stop to the harassment

08SX73; Blue: Rushed through very important questions leaving little to no time/opportunity for the patient to respond openly and therefore likely less than honestly. Provider engaged in an inappropriate and biased conversation/aside about the HPV vaccine. Overall, the interaction was cold and robotic from the provider with little to no regard/empathy for the patient. The provider failed to express the true importance and reason as to why the patient needed to sit and wait in the waiting area for 10 minutes post administration of the vaccines.

07HA64; Maroon: Patient was addressed in a quick and in a sense, disrespectful way. No time was given to the patient for their thoughts and concerns when the prescriber was attempting to resolve the problem. The prescriber’s words and tone kind of brushed off how the patient feels and how anxious they are.

**Station 2**

10NO66; Black: properly introduces themselves and welcomes patient to also
-emphasizes wanting to make patient comfortable
-maybe should have asked if they have stable housing before asking for an address
-actively addresses income/insurance concerns
-set expectations of visit for pt [care team, how long it’ll take, etc.]
-says “I understand, I know”, not great for motivational interviewing
-“viral load, hep C, sharps container” not patient friendly

Additional comment: - I think the scoring sheet is a little difficult to use as an evaluation technique for the station 1 and 2 since there is no “team”, rather it’s just the patient and a provider. For the future, it may be easier to have a recorded station 1 and 2 that we watch/evaluate that would include a team and their interactions similar to what we had for station 3.

30ZI70; Brown: Worked well with patient, worked around patient’s obstacles (their anxiety,
 financial/medical concerns, etc), very clear communication (what is to be done,
 what to expect, etc.), non-judgmental tone throughout (very understanding of patient’s concerns)

23EA62; Dark blue: Great relationship rapport in beginning – checks pronouns & name
Address was handled well – stability checked – email reminders
Trying to help her get insurance!
Asks why she is here – lays out plan before (xrays, how many ppl to expect, estimated time, asked for consent before starting) patient wants to be included in decisions
Sensitive about HIV, sharing needles, referral to Damion center, parts of body touching for trauma, let patient know it Is ok to touch them for a break.

Overall was amazing and they handled everything in a very sensitive and caring manner.

Additional comments: Thank you do much for this opportunity! I definitely learned a lot from this especially about interdisciplinary care with the SP and trauma-informed care.

06we67; White: This scenario was refreshing in that the doctor eases the anxiety of the patient while also giving them all the expectations of each step in the cleaning and screening during the appointment. This is how every appointment should go, as it provides the patient with ease and provides them with a calming experience that was once a terrifying one.

Additional comments: I appreciate there being two different scenarios rather than just one. This provides us with the ability to compare the two to see a great interaction with an uneasy interaction and how we should handle each patient with care and respect.

08SX73; Blue: Provider went above and beyond to establish a rapport and utilize collaborative communication with the patient. The provider explained everything that was going to happen before it happened and allowed the patient multiple opportunities to express their level of comfort with the appointment as well as to continue with the treatment. The provider established clear boundaries for what physical touch would occur and where they could be touched for the patient to express their level of comfort/discomfort. The provider gave multiple resources and referrals for additional resources that the patient could and should be accessing. Overall, the total interaction was patient centered and emphasized empowering the patient.

07HA64; Maroon: Good open-ended questions. Provides a safe space for the patient to talk. Asks patient for thoughts and concerns. The patient feels more relaxed and able to bring in their thoughts and concerns better.
